# The Ward Floor in All Materials

**Published:** 1912-04-06

**Authors:** 


### THE MAKING OF A MODERN HOSPITAL.*

### The Ward Floor in all Materials.

The problem of the ward floor is one that has exercised the minds of hospital architects considerably during the past half-century. Amidst the mass of speculation and theory, experience and experiment, one or two facts stand out very plainly. For example, it is now well established that the flooring of a ward is a matter of considerable importance so far as the purity of the air of the ward is concerned. Laveran, examining bacteriologically the air in a ward of a French general hospital, found on an average 16,000 germs per cubic metre present: a day later, when the floor had been scrubbed, he again analysed the air and found that the number of germs per c.m. had been more than doubled. The interstices and joints of the flooring, holding as they did an accumulation of débris and dust, are responsible for this augmentation in the bacteriological flora of the ward. Nor must it be imagined that the organisms discovered were all harmless. Laveran's researches proved that the air of a general ward, paved with deal boards, contained not only non-pyogenic bacteria, but a small percentage of very powerfully pathogenic germs, among which he isolated the bacilli of diphtheria, pneumonia, and tubercle, and the staphylococcus aureus and the streptococcus pyogenes longus. These findings agreed with the experience of hospital workers in the days when bacteriological examinations were unknown. At the Prague Hospital more than sixty years ago it was observed that there was apparently some connection between the sweeping of the ward floor and the incidence of severe cases of erisipelas and septicæmia.

### Old Burnt-brick Floors.

In the older hospitals, generally provided with floors of burnt brick, perhaps cemented over (the old Hôtel Dieu at Paris and the Holy Ghost Hospital at Rome were good examples of this style of flooring, which is still to be seen in a few old institutions, though not fortunately in this country), it was extremely difficult to secure the necessary cleanliness. In the modern hospital the first objects aimed at, so far as the floors are concerned, are to have smoothness without slipperiness; solidity, impermeability; no sharp corners, fissures, or crevices that can lodge dust and dirt; durability, and permanence. These are the primary essentials; to them we may reasonably add that the flooring must be capable of being easily and efficiently cleansed and it must not too readily absorb stains, that it must be unshrinkable, and that it must be economic. Taking all the varieties of floors which have been hitherto experimented with, it is safe to say that no single one can honestly claim to fulfil all these conditions or to possess all these essential qualities. Nevertheless it may be instructive briefly to summarise the findings with regard to the different styles of flooring.

### Wood Floors.

These are undoubtedly most common. Even in the newest hospitals some modification of the wood floor is to be found, and many authorities consider the wood floor, made of narrow planks of well-seasoned hard wood, waxed or oiled, as the ideal floor so far as durability and solidity are concerned. Nevertheless, it is incontestable that wood flooring, no matter how well laid and how carefully the planks may be dovetailed, is not impermeable, and in course of time shows narrow splits or tiny fissures which lodge bacteria-infected dirt. It is argued that by proper waxing and oiling such flooring is to all intents and purposes kept aseptic, but recent experiments at the St. Jean Hospital in Brussels have shown that waxing and oiling are not absolute safeguards. Wood flooring carelessly laid down is an abomination in the ward, a source of danger and expense which should not be tolerated for a moment. If a wood floor is decided on, the only permissible model is the narrow plank flooring of hard wood, laid down after the manner of a yacht's deck planking. The difficulty is to secure the planks. Usually they are dovetailed and nailed down, but nails, however carefully treated, invariably leave slight depressions or ridges which are difficult to keep clean. Any variety of hard wood is employed; the essentials are that the wood must be dry, well seasoned and shrunk, and carefully laid down. Maple or teak is probably the best, though both show a tendency to shrink, especially when frequently oiled. All wood floors must be frequently waxed, and most of them are much improved by the application of a dustless oil. When well laid and carefully attended to, a wood floor greatly adds to the appearance of the ward; it is undoubtedly one of the handsomest varieties of floorings, but it can scarcely be said to be the cheapest or least permeable. Its advantages are that it is easy to keep clean, hard wearing, practically non-slipping and warm. One of its great objections is that it renders the rounding of the angles made by the floor with the walls very difficult.

Parquet flooring is really a modification of wood flooring, and should properly be classed in the former group. It is customary, however, to deal with it as a separate class. A great variety of styles of the parquet can be seen in the newer hospitals, where this kind of floor has been extensively tried, though, owing chiefly to its costliness, not so much in the wards as in the administrative block. The advantages of the parquet floor are that it permits of greater closeness of apposition, that it permits of a faulty or worn portion of flooring being easily replaced by new without disturbing the whole floor, that it is very durable, slightly more impermeable than ordinary wood flooring, and that it is undoubtedly a very handsome-looking flooring. With care the parqueting can be carried some distance

* Previous articles have appeared in The Hospital of Oct. 7, 14, 21, 28, Nov. 4, 18, Dec. 9, 30, Feb. 10, and March 16.

up the wall so as to round the floor angles, as is the case in the new wards at the Johns Hopkins' and in the Children's Hospital at St. Petersburg. The main objections to parquet floors are that they are at present expensive—certainly much more so than a first-class wood floor—that in the method in which each block is nailed down a large number of nail depressions are made; that such flooring cannot very well be washed every day, but must be treated with oil and wax, the result of which is that a parquet floor is generally slippery and needs a certain amount of powder (an aid which is not permissible in a hospital ward); and finally, that unless the parqueting is carried on to the walls the floor angle must be rounded by a plinth, which introduces further crevices and opportunities for the lodgment of dust. On the whole, however, the parquet floor is a very suitable style of ward flooring, and coming into greater favour as its construction becomes cheaper.

The main varieties of the parquet floor are the hard wood parquet block method which is general in England and America. The most popular wood is undoubtedly teak; maple blocks, which are less expensive, are employed in some institutions; oak blocks, with a more open fibre than either teak or maple, are unsuitable for ward floors. On the Continent various other methods are used. The dovetailed parquet, in which the blocks are laid down on a thin wood floor, is rarely used in institutions; it is very expensive and quite unsuitable for ward use. The methods preferred are those in which the blocks are nailed down, or laid in a matrix of tar or cement. Both these are excellent, but have the disadvantage that renewal of a part of the flooring is more difficult than with the English method. Parqueting by means of blocks made of compressed wood fibre, paper, or lithoxyle are unsuitable for hospital use.

### Linoleum Flooring.

Undoubtedly one of the most satisfactory and one of the relatively cheap forms of flooring for hospital wards is linoleum. In older hospitals linoleum was used not as a flooring by itself but as a floor covering, largely to deaden sound. In the newer institutions, however, its value as a flooring by itself has been amply demonstrated. It is best laid on a thin cement foundation. It is easily cleaned, relatively impermeable, on the whole very durable, and in point of view of expense compares favourably with all other floors.

### Matrix Floors.

Of this type are the ordinary cement floor which by itself is no longer used, though as a foundation for linoleum or composition flooring it is still employed; the terrazzo floor, which is unsuitable for the ward, although it answers very well for the hall and certain portions of the annexes; the mosaic floor, of which there are two principal varieties, and their modifications. All these are unsuitable for the ward, because they are slippery, especially when washed, very cold, noisy, and, above all, liable to crack. In the operating theatres, corridors, and lobbies, which can be swept by the hose, they are to be recommended, but even here their tendency to split and crack makes them unsuitable if a tile floor can be had. Even in new hospitals where the most elaborate precautions have been taken to prevent cracks in the terrazzo flooring, as at Eppendorf, the new wards of the Moabite and the Roosevelt Hospital, cracks are very apparent in the flooring from time to time. A matrix floor is certainly not to be advised for a general ward.

### Tiled Floors.

These have come into favour chiefly in France and Belgium, while a few of the newer German clinics have also adopted them. They have the advantage of being durable, perfectly impermeable, easily cleaned, and very strong. On the other hand, they are very cold and, unless specially roughened or studded, dangerous by reason of their excessive slipperiness. Formerly another great objection to them was the fact that they were laid down in cement matrix; the result was that the cement between the tiles got eroded, leaving a crevice or fissure. Now, however, they are invariably fixed in a special composition, generally Porcelankit, which is smoothed down flush with the tile so that there is left no angle, fissure, or crevice. Metallic tiles have also been employed; but they have several objections and are not likely to become popular. Tiled floors are excellent for the operating theatres, lecture rooms, lobbies, central halls, and bathrooms. They are unsuitable for the ward, chiefly because of their coldness, and if used in a ward must, like the matrix floor, be covered with linoleum. Another disadvantage is that they are very expensive. The best German tiles are very good, but their price is such that a tiled floor is about half as expensive again as a wood floor. French and Belgian tiles are less costly, but are said to be less durable. Lately some hospitals have tentatively experimented with india-rubber tiles. These are very durable, clean, comfortable to the feet, and make an excellent flooring. They have been laid down with very satisfactory results in several public buildings—notably in the White Star buildings at Liverpool—but they are still so expensive that their cost is almost prohibitive in a general ward.

### Composition Floors.

Many varieties of these are on the market and in former issues of The Hospital we have already briefly referred to some of them. Among those which experience abroad has found most satisfactory are the composition flooring of which the basis is magnesia incorporated with resin and cement and a variable amount of sawdust. One of the best known of these is "Torgament," which has been widely tried, with excellent results, in the newer Belgian clinics. On the other hand, many authorities state that this flooring is very brittle, slippery, and liable to crack with variations of temperature. Prismalite, porphyrolite, litholine, xylopalm, dolomemt, calcinite, asphalto, dielol, lapidol, and various other compositions have been tried, but the opinions expressed are so diverse that all that can be stated at present is that a composition flooring offers no advantages over wood or parquet.

